# Influenza Vaccination in Pregnant Women: A Systematic Review

**DOI:** 10.5402/2013/879493

**Published:** 2013-11-07

**Authors:** Tais F. Galvao, Marcus T. Silva, Ivan R. Zimmermann, Luiz Antonio B. Lopes, Eneida F. Bernardo, Mauricio G. Pereira

**Affiliations:** ^1^Faculty of Medicine, University of Brasilia, Campus Universitario, Conj 16, Sala 77, 70904-970 Brasilia, DF, Brazil; ^2^Getulio Vargas University Hospital, Federal University of Amazonas, Rua Apurina 4, Centro, 69020-170 Manaus, AM, Brazil; ^3^Faculty of Medicine, Federal University of Amazonas, Rua Afonso Pena 1053, Centro, 69020-160 Manaus, AM, Brazil; ^4^State Health Department, LACEN, Setor de Areas Isoladas Norte, Bloco B, 70086-900 Brasilia, DF, Brazil

## Abstract

*Objective*. To assess the effects of the inactivated influenza virus vaccine on influenza outcomes in pregnant women and their infants. *Methods*. We performed a systematic review of the literature. We searched for randomized controlled trials and cohort studies in the MEDLINE, Embase, and other relevant databases (inception to September 2013). Two researchers selected studies and extracted the data independently. We used the Grading of Recommendations Assessment, Development, and Evaluation (GRADE) approach to assess the quality of the evidence. *Results*. We included eight studies out of 1,967 retrieved records. Influenza vaccination in pregnant women significantly reduced the incidence of influenza-like illness in mothers and their infants when compared with control groups (high-quality evidence) and reduced the incidence of laboratory-confirmed influenza in infants (moderate-quality evidence). No difference was found with regard to influenza-like illness with fever higher than 38°C (moderate-quality evidence) or upper respiratory infection (very-low-quality evidence) in mothers and infants. *Conclusions*. Maternal vaccination against influenza was shown to prevent influenza-like illness in women and infants; no differences were found for other outcomes. As the quality of evidence was not high overall, further research is needed to increase confidence and could possibly change these estimates.

## 1. Introduction

Pregnant women and neonates are at greater risk for influenza-related complications than the general population [1, 2]. Most institutions and organizations recommend that all pregnant women receive the trivalent inactivated influenza virus vaccine [3–8]. Such endorsements rely on the immunogenic response of the mothers, the lack of teratogenicity, and the contrandication of immunization in children younger than six months [7, 9–11].

Despite the broad recommendation to vaccinate pregnant women against influenza, coverage is still limited. A survey held by the Centers for Disease Control and Prevention involving women who were pregnant from October 2011 to January 2012 showed that only half of the respondents had been vaccinated and fewer than 10% had received the vaccine before giving birth [12]. Similar patterns were found in previous studies [13].

Although there is clear evidence of the efficacy of the influenza vaccine for the general population [14], to our knowledge, a systematic approach with regard to the evidence of the therapeutic effects of influenza vaccination in pregnant women is lacking. Our objective is to review the effects of influenza vaccination in preventing influenza-related outcomes in pregnant women and their infants.

## 2. Methods

### 2.1. Eligibility Criteria for the Included Studies

We selected randomized controlled trials or cohort studies that assessed the effects of inactivated influenza vaccine in preventing influenza-related outcomes in pregnant women and their offspring compared with placebo, other vaccines, or no vaccines. We excluded studies that assessed monovalent vaccines, such as the H1N1 influenza vaccine, because they are used for specific epidemic situations.

### 2.2. Data Sources and Search Strategy

We searched for eligible studies in the following databases (from inception to September 2013): MEDLINE, Embase, Scopus, Centre for Reviews and Dissemination (CRD), Cochrane Central Register of Controlled Trials (CENTRAL), metaRegister of Current Controlled Trials (mCRT), Latin American and Caribbean Center on Health Sciences Information (LILACS), and Scientific Electronic Library Online (SciELO). References to relevant publications in the field were also screened to identify potentially eligible studies. There were no restrictions on language, length of followup, publication date, or publication status. Those databases comprise the main sources of cohort studies and clinical trials.

We used the following search terms to search MEDLINE (via PubMed) and adapted the strategy for the other databases: (“influenza, human”[mesh] or “influenza”[tiab] or “human flu”[tiab] or “influenza”[tiab] or “influenzas”[tiab] or “grippe”[tiab] or “flu”[tiab] or “cold”[tiab]) and (“influenza vaccines”[mesh] or “influenza vaccines”[tiab] or “vaccine”[tiab] or “vaccine”[tiab] or “vaccines”[tiab]) and (“mothers”[mesh] or “mothers”[tiab] or “pregnancy”[mesh] or “pregnancy”[tiab] or “gestation”[tiab] or “pregnant women”[mesh] or “pregnant women”[tiab] or “pregnant”[tiab]) and (“infant”[mesh] or “infant”[tiab] or “infants”[tiab] or “infant, newborn”[mesh] or “newborn”[tiab] or “newborns”[tiab] or “fetus”[mesh] or “fetus”[tiab] or “foetus”[tiab] or “fetal”[tiab] or pregnancy).

### 2.3. Study Selection and Data Collection Process

Two independent reviewers (EB and LABL) selected the studies by assessing titles and abstracts and extracted the data. Disagreements were resolved by consensus or a third reviewer (TFG). The extracted data consisted of the following: year, country, study design, gestational age, type of vaccine, posology, comparators, sample size, followup, and outcomes. When necessary, we contacted the corresponding authors for additional information.

### 2.4. Risk of Bias and Quality Assessment

To assess the risk of bias of randomized controlled trials (RCT), we used the Cochrane Collaboration tool [15], which includes judgments about the sequence generation, allocation sequence concealment, blinding, incomplete outcome data, selective outcome reporting, and other sources of bias. For observational studies, we evaluated the following: eligibility criteria, measurements of exposures and outcomes, control of confounding, and followup [16].

We assessed the quality of evidence for each relevant outcome with the Grading of Recommendations Assessment, Development, and Evaluation (GRADE) [17, 18]. For this evaluation, we separated the bodies of evidence into “experimental” and “observational” centered on RCT and cohort studies, respectively. Following the GRADE approach, RCT started the evaluation with “high quality” and cohort studies with low quality of evidence. Then, we assessed the evidence against five items that could decrease its quality: limitations (risk of bias), inconsistency, indirectness, imprecision, and publication bias. After assessing these items, the resulting quality of evidence could be rated as high, moderate, low, or very low. When distinct levels of quality were available for the same outcome, we considered the experimental design (RCT) evidence in rating the quality.

The final judgments regarding the risk of bias and evidence quality were achieved by consensus. We considered the quality assessment results when interpreting the findings.

### 2.5. Data Analysis

The primary outcome was the incidence of influenza-like illness, which was defined as fever and either cough or sore throat. For infants' outcomes, we defined small for gestational age as a weight below the 10th percentile and prematurity as birth before a gestational age of 37 complete weeks. 

We extracted the estimates along with 95% confidence intervals (95% CI) according to the data available in the original studies (relative risk (RR), odds ratio (OR), or hazard ratio (HR)). If reported, we only considered the adjusted estimates and did not perform further calculation. We attempted to perform meta-analyses using random effects models, if numerical data from studies allowed a summarization.

## 3. Results

**Figure 1:**
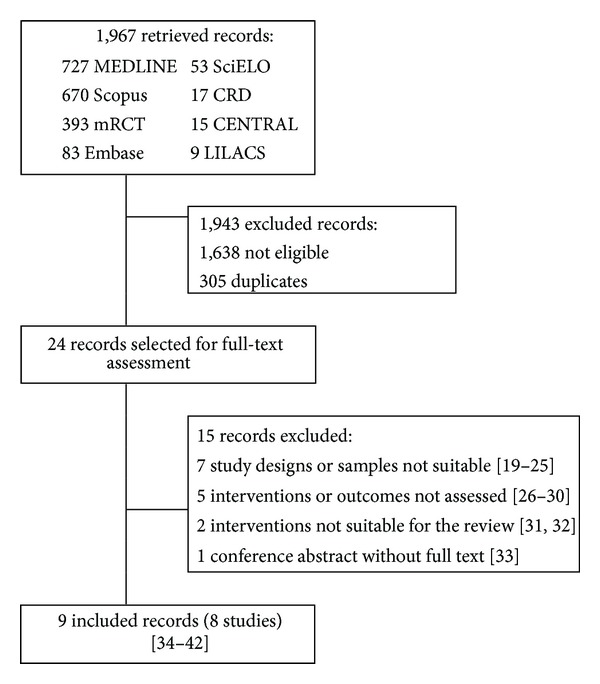
The results of the search, selection and inclusion of studies.

Our search retrieved 1,967 records. Twenty-three records were selected for full-text assessment, and nine were included in the review. The reasons for exclusions are depicted in Figure 1 [19–33]. were related to eight unique studies that enrolled 182,820 pregnant women and 182,246 neonates [34–42]. 

### 3.1. Study Characteristics

Table 1 describes the main characteristics of the included studies. Except for one, all studies were published from 1990 to 2012. We identified one RCT conducted in Bangladesh and seven cohort studies performed in the United States.

The trivalent inactivated vaccine was the most common intervention and was assessed in seven studies [35–41]. One cohort used the polyvalent inactivated vaccine [34]. Newborns were not vaccinated. Only the RCT had an active control group (pneumococcal vaccine) [38]. Nearly the entire sample of pregnant women came from three retrospective cohorts [35, 37, 41]. The data in the retrospective studies came from medical records. The length of followup of each outcome varied among studies and lasted up to 36 weeks. Some studies adjusted their results for confounding factors, such as the women's age, week of delivery, infant's gender, and gestational age.

Observational studies that compared baseline characteristics of the groups showed that most variables did not differ between the groups. Some studies showed that vaccinated women had a worse profile than unvaccinated women, which were of higher risk for complications [37], were older, and had higher body mass index, higher parity, and more multiple gestation [42]. One study observed that vaccinated mothers had more health insurance than unvaccinated ones [41]. All studies controlled the identified confounding through multivariable analysis.

### 3.2. Outcomes and Quality of Evidence

We could not perform meta-analysis because the studies used different measures of association for the same outcome; the estimates are presented as available in the studies.

Table 2 depicts the results of each outcome and the quality assessment. Mothers who received the influenza vaccine had a lower incidence of influenza-like illness, as did their infants (high-quality evidence), but there was no difference found for influenza-like illness with fever higher than 38°C (moderate-quality evidence). A lower incidence of influenza in infants, as confirmed by laboratory tests, was also observed (moderate-quality evidence).

Very-low-quality evidence showed no difference between comparisons with regard to the incidence of upper respiratory infection, hospitalization, and medical visits for influenza-like illness in mothers and infants.

Two studies found no difference in the incidence of hospitalization for influenza-like illness in infants [35, 37]; in one study, the reduction in the rate of hospital admission was significant [40]. With regard to medical visits for influenza-like illness in infants, the observational studies showed no significant differences [35, 37, 40], and the RCT showed a reduction in such rate [38] (moderate-quality evidence).

For the outcomes prematurity and small for gestational age, conflicting results were found across the studies. One single cohort [42] indicated significant reduction in stillbirth and neonatal death among the influenza-vaccinated group (moderate-quality evidence).

We did not assess the incidence of adverse reactions because the included studies did not systematically evaluate this outcome. In general, influenza vaccination had no association with local or minor systemic effects, fever, Apgar score at one minute, hyperbilirubinemia, or major malformations.

## 4. Discussion

The influenza vaccine was found to reduce the risk of influenza-like illness in mothers and infants as well as the risk of laboratory-confirmed influenza in infants. Such findings are supported by high- and moderate-quality evidence. Other outcomes showed no significant differences between the compared groups. Adverse reactions were not systematically assessed across the studies, but there was no evidence of increase in clinically relevant risk related to influenza vaccination during pregnancy. A big cohort study that focused on the safety of trivalent inactivated influenza vaccine, however, did not find any increased risk of adverse events and adverse obstetric events in the vaccinated mothers, when compared to unvaccinated pregnant women [43, 44].

Other factors can prevent influenza in infants such as the effect of breast-feeding [45] and immunization of all the infant's close contacts, also known as cocooning [46]. To avoid confounding and enable comparison, groups should be set by randomization. In the present review, however, only one RCT was identified and included. Although most observational studies performed multivariate analyses, residual confounding may remain even after adjustment because this statistical procedure cannot control for all biological variabilities [47].

Controversy may rise about the possible differences between the groups from observational studies. Women receiving influenza vaccine would have more medical attention and be healthier than unvaccinated pregnant women; thus, the result found would be attributed to the health profile of vaccinated women rather than to the vaccine itself. However, some studies reported that vaccinated women were at high risk during gestation, and this difference was also statistically controlled [37, 42]. Studies consistently reported that the patients and the clinicians made the decision about taking influenza vaccine or not. Surveys about attitudes and beliefs of these actors regarding influenza vaccination in pregnancy show that the proportion of people who do not believe vaccine is safe is still high [48, 49].

Another limitation of our review is the absence of the systematic reporting of adverse reactions. Although the incidence of adverse reaction was not shown to be a concern in the included studies, this lack of evidence may inadvertently lead to the conclusion that this risk is minimal or nonexistent [50]. Individuals with egg allergy, for example, require caution when receiving trivalent inactivated influenza vaccine [7].

Previous narrative reviews concluded that influenza vaccination is safe, that there has been no evidence of teratogenicity, and that many countries recommend influenza vaccination among women with both healthy and high-risk pregnancies [9, 51–56]. The role of education of patients and doctors in increasing adherence to maternal vaccination was also emphasized [57–59]. One systematic review assessed the benefits and dangers of the influenza vaccine in special populations—pregnant women included—but limited the eligible studies to RCTs only [60]. Another study reviewed the beneficial effects on the influenza vaccine on infants only [61].

Some barriers still persist to the implementation of influenza vaccine during pregnancy. Apprehensions about the use of thimerosal-containing influenza vaccines, based on theoretical risk of harm to the fetal brain, were widely spread in scientific and lay communities during the past years [55, 62]. Subsequent research proved that no causal relation existed between immunization with vaccine containing thimerosal preservative—including exposure during pregnancy—and neuropsychological outcomes [63, 64]. The most recent report of the Global Advisory Committee on Vaccine Safety of the World Health Organization considered that available evidence strongly supports the safety of the use of thimerosal as a preservative for inactivated vaccines [65]. It is expected that with the availability of higher-quality evidence, such concerns can be demystified.

Attending to claims for more evidence [66, 67], several RCTs assessing influenza vaccination in pregnancy are planned and some are ongoing [68–77]. Such RCTs focus on different populations, such as HIV-positive mothers, and factors that interfere with immunization coverage. We expect that, following the publication of these trials, the availability and quality of the evidence will radically improve. Additionally, the issue about the comparability between the vaccinated and unvaccinated groups will be more properly addressed.

## 5. Conclusion

Maternal immunization for influenza significantly reduced the incidence of influenza-like illness in women and infants. For clinical practice, the findings reinforce the current recommendations to vaccinate all pregnant women against influenza. We are not confident in making conclusions about other outcomes. Further studies should address this lack of evidence and enhance the overall quality of the outcomes.

## Figures and Tables

**Table 1:** Main characteristics of the included studies.

Study	Period of enrollment	Country	Study design	Influenza vaccine group (*N*)	Control group (*N*)	Gestational month at immunization
Hulka 1964 [34]	1962-1963	USA	Prospective cohort	Polyvalent inactivated (363)	Placebo (181)	>3rd*
Black et al. 2004 [35]	1997–2002	USA	Retrospective cohort	Trivalent inactivated (3,707)	No vaccine (45,878)	7th–9th
Munoz et al. 2005 [36]	1998–2003	USA	Matched retrospective cohort	Trivalent inactivated (225)	No vaccine (826)	4th–9th
France et al. 2006 [37]	1995–2001	USA	Matched retrospective cohort	Trivalent inactivated (3,160)	No vaccine (37,969)	4th–9th
Zaman et al. 2008 [38, 39]	2004-2005	Bangladesh	Randomized controlled trial	Trivalent inactivated (172)	Pneumococcal vaccine (168)	7th–9th
Eick et al. 2011 [40]	2002–2005	USA	Prospective cohort	Trivalent inactivated (573)	No vaccine (587)	4th–9th
Omer et al. 2011 [41]	2004–2006	USA	Retrospective cohort	Trivalent inactivated (578)	No vaccine (3,590)	1st–9th
Sheffield et al. 2012 [42]	2003–2008	USA	Retrospective cohort	Trivalent inactivated (8,690)	No vaccine (76,153)	1st–9th^†^

*Data assumed by the authors from information available in the paper.

^†^From October 2003 through March 2004, women were vaccinated in the second and third trimesters. From October 2004 through March 2008, women were vaccinated in all three trimesters.

*N*: number of pregnant women in each group.

USA: United States of America.

**Table 2:** Outcomes and the quality of evidence of influenza vaccination in pregnant women.

Outcome	Population	Study	Followup (weeks)	Measure of association	Result (95% CI)	Quality of evidence
Influenza-like illness	Mothers	Hulka 1964 [34]	12 to 30	RR	0.56 (0.35–0.91)^†^	High
		Zaman et al. 2008 [38]	24 to 36*	IRR	0.64 (0.43–0.96)^†^	
	Infants	Zaman et al. 2008 [38]	24	IRR	0.71 (0.54–0.93)^†^	High

Influenza-like illness with fever >38°C	Mothers	Zaman et al. 2008 [38]	24 to 36*	IRR	0.57 (0.30–1.09)^†^	Moderate^a^
	Infants	Zaman et al. 2008 [38]	24	IRR	0.72 (0.49–1.05)^†^	Moderate^a^

Laboratory-confirmed influenza	Infants	Zaman et al. 2008 [38]	24	IRR	0.37 (0.15–0.95)^†^	Moderate^a^
		Eick et al. 2011 [40]	26	RR	0.59 (0.37–0.93)	

Acute upper respiratory tract infection	Mothers	Munoz et al. 2005 [36]	1.1 to 26	RR	1.84 (0.87–3.87)^†^	Very low^a,b^
	Infants	Munoz et al. 2005 [36]	5 to 26	RR	1.13 (0.87–1.44)^†^	Very low^a,b^
		France et al. 2006 [37]	≤13	RR	0.83 (0.64–1.08)^†^	

Hospitalization for influenza-like illness	Mothers	Munoz et al. 2005 [36]	6	RR	3.67 (0.23–58.47)^†^	Very low^a,b^
	Infants	Black et al. 2004 [35]	≤17	HR	0.63 (0.30–1.29)	Very low^a,b,c^
		Munoz et al. 2005 [36]	1.1 to 26	RR	3.73 (0.23–59.39)^†^	
		France et al. 2006 [37]	≤13	RR	1.39 (0.42–4.58)^†^	
		Eick et al. 2011 [40]	26	RR	0.61 (0.45–0.84)	

Medical visit for influenza-like illness	Mothers	Black et al. 2004 [35]	16 or less	HR	1.00 (0.84–1.20)	Moderate^a^
		Munoz et al. 2005 [36]^†^	1.1 to 26	RR	1.35 (1.02–1.78)^†^	
		Zaman et al. 2008 [38]	24 to 36*	IRR	0.75 (0.39–1.44)^†^	
	Infants	Black et al. 2004 [35]	≤17	HR	0.96 (0.89–1.03)	Moderate^a^
		France et al. 2006 [37]	≤13	RR	0.96 (0.86–1.07)	
		Zaman et al. 2008 [38]	24	IRR	0.58 (0.41–0.82)^†^	
		Eick et al. 2011 [40]	26	RR	0.92 (0.73–1.16)	

Prematurity	Infants	Black et al. 2004 [35]	At birth	RR	1.10 (0.97–1.23)^†^	Very low^a,b^
		Munoz et al. 2005 [36]	At birth	OR	0.67 (0.32–1.32)	
		Zaman et al. 2008 [38, 39]	At birth	OR	0.48 (0.08–2.74)	Moderate^a^
		Omer et al. 2011 [41]^‡^	At birth	OR	0.60 (0.38–0.94)	
		Sheffield et al. 2012 [42]^†^	At birth	OR	0.86 (0.78–0.95)	

Small for gestational age	Infants	Zaman et al. 2008 [38, 39]	At birth	OR	0.44 (0.19–0.99)	Moderate^a^
		Omer et al. 2011[41]^‡^	At birth	OR	0.74 (0.47–1.15)	
		Sheffield et al. 2012 [42]^†^	At birth	OR	1.00 (0.93–1.07)	

Stillbirths	Infants	Sheffield et al. 2012 [42]^†^	All months	OR	0.61 (0.42–0.88)	Moderate^a^

Neonatal death	Infants	Sheffield et al. 2012 [42]^†^	All months	OR	0.55 (0.35–0.88)	Moderate^a^

IRR: incidence rate ratio; RR: relative risk; HR: hazard ratio; OR: odds ratio.

Notes: *women were followed during pregnancy, beginning at two weeks after vaccination until delivery and continuing for 24 weeks after delivery. ^†^Data calculated by the authors from information available in the paper. ^‡^Outcome measured at the peak of the influenza season.

Reasons for rating down the on-the-quality assessment using the GRADE approach.

^a^Serious imprecision: low number of events.

^b^The study design was observational.

^c^Serious inconsistency: results vary greatly across studies.

## References

[1] Lai J, Fay KE, Bocchini JA (2011). Update on childhood and adolescent immunizations: selected review of US recommendations and literature: part 2. *Current Opinion in Pediatrics*.

[2] Rasmussen SA, Jamieson DJ, Uyeki TM (2012). Effects of influenza on pregnant women and infants. *American Journal of Obstetrics and Gynecology*.

[3] C. D. C. Advisory Committee on Immunization Practices Centers for Disease Control and Prevention (2008). Guiding principles for development of ACIP recommendations for vaccination during pregnancy and breastfeeding. *Morbidity and Mortality Weekly Report*.

[4] World Health Organization WHO Sex, gender and influenza. http://whqlibdoc.who.int/publications/2010/9789241500111_eng.pdf.

[5] A. C. O. G. American College of Obstetricians and Gynecologists Committee on Obstetric Practice (2010). Committee Opinion No. 468: influenza vaccination during pregnancy. *Obstetrics & Gynecology*.

[6] N. C. I. R. S. National Centre for Immunisation Research & Surveillance Influenza vaccines for Australians: information for immunisation providers. http://www.ncirs.edu.au/immunisation/fact-sheets/influenza-fact-sheet.pdf.

[7] C. D. C. Centers for Disease Control and Prevention (2012). Prevention and control of influenza with vaccines: recommendations of the Advisory Committee on Immunization Practices (ACIP)—United States, 2012–13 influenza season. *Morbidity and Mortality Weekly Report (MMWR)*.

[8] European Centre for Disease Prevention and Control. http://www.ecdc.europa.eu/en/publications/Publications/Seasonal%20influenza%20vaccination%20of%20children%20and%20pregnant%20women.pdf.

[9] Tamma PD, Steinhoff MC, Omer SB (2010). Influenza infection and vaccination in pregnant women. *Expert Review of Respiratory Medicine*.

[10] Munoz FM (2012). Safety of influenza vaccines in pregnant women. *American Journal of Obstetrics and Gynecology*.

[11] Bednarczyk RA, Adjaye-Gbewonyo D, Omer SB (2012). Safety of influenza immunization during pregnancy for the fetus and the neonate. *American Journal of Obstetrics and Gynecology*.

[12] C. D. C. Centers for Disease Control and Prevention (2012). Influenza vaccination coverage among pregnant women—2011–12 influenza season, United States. *Morbidity and Mortality Weekly Report*.

[13] Kennedy ED, Ahluwalia IB, Ding H, Lu PJ, Singleton JA, Bridges CB (2012). Monitoring seasonal influenza vaccination coverage among pregnant women in the United States. *American Journal of Obstetrics and Gynecology*.

[14] Osterholm MT, Kelley NS, Sommer A, Belongia EA (2012). Efficacy and effectiveness of influenza vaccines: a systematic review and meta-analysis. *The Lancet Infectious Diseases*.

[15] Higgins J, Green S Cochrane Handbook for Systematic Reviews of Interventions. http://www.cochrane-handbook.org.

[16] Meerpohl JJ, Langer G, Perleth M, Gartlehner G, Kaminski-Hartenthaler A, Schünemann H (2012). [GRADE guidelines: 4. Rating the quality of evidence—limitations of clinical trials (risk of bias)]. *Zeitschrift für Evidenz, Fortbildung und Qualität im Gesundheitswesen*.

[17] Schünemann H, Bro?ek J, Oxman A GRADE handbook for grading quality of evidence and strength of recommendation. Version 3. 2. http://ims.cochrane.org/gradepro.

[18] Balshem H, Helfand M, Schünemann HJ, Oxman AD, Kunz R, Brozek J (2011). GRADE guidelines: 3. Rating the quality of evidence. *Journal of Clinical Epidemiology*.

[19] Heinonen OP, Shapiro S, Monson RR, Hartz SC, Rosenberg L, Slone D (1973). Immunization during pregnancy against poliomyelitis and influenza in relation to childhood malignancy. *International Journal of Epidemiology*.

[20] Sumaya CV, Gibbs RS (1979). Immunization of pregnant women with influenza A/New Jersey/76 virus vaccine: reactogenicity and immunogenicity in mother and infant. *The Journal of Infectious Diseases*.

[21] Englund JA, Mbawuike IN, Hammill H, Holleman MC, Baxter BD, Glezen WP (1993). Maternal immunization with influenza or tetanus toxoid vaccine for passive antibody protection in young infants. *The Journal of Infectious Diseases*.

[22] Yeager DP, Toy EC, Baker B (1999). Influenza vaccination in pregnancy. *American Journal of Perinatology*.

[23] Tuyishime JD, De Wals P, Moutquin JM, Frost E (2003). Influenza-like illness during pregnancy: results from a study in the eastern townships, Province of Quebec. *Journal of Obstetrics & Gynaecology*.

[24] Benowitz I, Esposito DB, Gracey KD, Shapiro ED, Vázquez M (2010). Influenza vaccine given to pregnant women reduces hospitalization due to influenza in their infants. *Clinical Infectious Diseases*.

[25] Poehling KA, Szilagyi PG, Staat MA (2011). Impact of maternal immunization on influenza hospitalizations in infants. *American Journal of Obstetrics and Gynecology*.

[26] Griffiths PD, Ronalds CJ, Heath RB (1980). A prospective study of influenza infections during pregnancy. *Journal of Epidemiology and Community Health*.

[27] Singleton JA, Lloyd JC, Mootrey GT, Salive ME, Chen RT (1999). An overview of the vaccine adverse event reporting system (VAERS) as a surveillance system. *Vaccine*.

[28] Irving WL, James DK, Stephenson T (2000). Influenza virus infection in the second and third trimesters of pregnancy: a clinical and seroepidemiological study. *British Journal of Obstetrics and Gynaecology*.

[29] Dodds L, McNeil SA, Fell DB (2007). Impact of influenza exposure on rates of hospital admissions and physician visits because of respiratory illness among pregnant women. *Canadian Medical Association Journal*.

[30] Orozova-Bekkevold I, Jensen H, Stensballe L, Olsen J (2007). Maternal vaccination and preterm birth: using data mining as a screening tool. *Pharmacy World and Science*.

[31] Murray DL, Imagawa DT, Okada DM, St. Geme JW (1979). Antibody response to monovalent A/New Jersey/8/76 influenza vaccine in pregnant women. *Journal of Clinical Microbiology*.

[32] Deinard AS, Ogburn P (1981). A/NJ/8/76 influenza vaccination program: effects on maternal health and pregnancy outcome. *American Journal of Obstetrics and Gynecology*.

[33] Guo Y, Allen V, Bujold E, Coleman B, Drews S, Gouin K (2010). Efficacy and safety of pandemic influenza vaccine in pregnancy. *Canadian Journal of Infectious Diseases and Medical Microbiology*.

[34] Hulka JF (1964). Effectiveness of polyvalent influenza vaccine in pregnancy. Report of A. *Obstetrics and Gynecology*.

[35] Black SB, Shinefield HR, France EK, Fireman BH, Platt ST, Shay D (2004). Effectiveness of influenza vaccine during pregnancy in preventing hospitalizations and outpatient visits for respiratory illness in pregnant women and their infants. *American Journal of Perinatology*.

[36] Munoz FM, Greisinger AJ, Wehmanen OA (2005). Safety of influenza vaccination during pregnancy. *American Journal of Obstetrics and Gynecology*.

[37] France EK, Smith-Ray R, McClure D (2006). Impact of maternal influenza vaccination during pregnancy on the incidence of acute respiratory illness visits among infants. *Archives of Pediatrics and Adolescent Medicine*.

[38] Zaman K, Roy E, Arifeen SE (2008). Effectiveness of maternal influenza immunization in mothers and infants. *The New England Journal of Medicine*.

[39] Steinhoff MC, Omer SB, Roy E (2012). Neonatal outcomes after influenza immunization during pregnancy: a randomized controlled trial. *Canadian Medical Association Journal*.

[40] Eick AA, Uyeki TM, Klimov A (2011). Maternal influenza vaccination and effect on influenza virus infection in young infants. *Archives of Pediatrics and Adolescent Medicine*.

[41] Omer SB, Goodman D, Steinhoff MC (2011). Maternal influenza immunization and reduced likelihood of prematurity and small for gestational age births: a retrospective cohort study. *PLoS Medicine*.

[42] Sheffield JS, Greer LG, Rogers VL, Roberts SW, Lytle H, McIntire DD (2012). Effect of influenza vaccination in the first trimester of pregnancy. *Obstetrics & Gynecology*.

[43] Nordin JD, Kharbanda EO, Benitez GV, Nichol K, Lipkind H, Naleway A (2013). Maternal safety of trivalent inactivated influenza vaccine in pregnant women. *Obstetrics & Gynecology*.

[44] Kharbanda EO, Vazquez-Benitez G, Lipkind H, Naleway A, Lee G, Nordin JD (2013). Inactivated influenza vaccine during pregnancy and risks for adverse obstetric events. *Obstetrics & Gynecology*.

[45] Chantry CJ, Howard CR, Auinger P (2006). Full breastfeeding duration and associated decrease in respiratory tract infection in US children. *Pediatrics*.

[46] Grizas AP, Camenga D, Vázquez M (2012). Cocooning: a concept to protect young children from infectious diseases. *Current Opinion in Pediatrics*.

[47] Sainani K (2011). The limitations of statistical adjustment. *PM and R*.

[48] Broughton DE, Beigi RH, Switzer GE, Raker CA, Anderson BL (2009). Obstetric health care workers’ attitudes and beliefs regarding influenza vaccination in pregnancy. *Obstetrics and Gynecology*.

[49] Lu AB, Halim AA, Dendle C (2012). Influenza vaccination uptake amongst pregnant women and maternal care providers is suboptimal. *Vaccine*.

[50] Alderson P (2004). Absence of evidence is not evidence of absence. *British Medical Journal*.

[51] Goldman RD, Koren G (2002). Motherisk Update: influenza vaccination during pregnancy. *Canadian Family Physician*.

[52] Englund JA (2003). Maternal immunization with inactivated influenza vaccine: rationale and experience. *Vaccine*.

[53] Naleway AL, Smith WJ, Mullooly JP (2006). Delivering influenza vaccine to pregnant women. *Epidemiologic Reviews*.

[54] Mak TK, Mangtani P, Leese J, Watson JM, Pfeifer D (2008). Influenza vaccination in pregnancy: current evidence and selected national policies. *The Lancet Infectious Diseases*.

[55] Tamma PD, Ault KA, del Rio C, Steinhoff MC, Halsey NA, Omer SB (2009). Safety of influenza vaccination during pregnancy. *American Journal of Obstetrics and Gynecology*.

[56] Jamieson DJ, Kissin DM, Bridges CB, Rasmussen SA (2012). Benefits of influenza vaccination during pregnancy for pregnant women. *American Journal of Obstetrics and Gynecology*.

[57] MacDonald NE, Riley LE, Steinhoff MC (2009). Influenza immunization in pregnancy. *Obstetrics and Gynecology*.

[58] Blanchard-Rohner G, Siegrist C-A (2011). Vaccination during pregnancy to protect infants against influenza: why and why not?. *Vaccine*.

[59] Shavell VI, Moniz MH, Gonik B, Beigi RH (2012). Influenza immunization in pregnancy: overcoming patient and health care provider barriers. *American Journal of Obstetrics and Gynecology*.

[60] Michiels B, Govaerts F, Remmen R, Vermeire E, Coenen S (2011). A systematic review of the evidence on the effectiveness and risks of inactivated influenza vaccines in different target groups. *Vaccine*.

[61] Steinhoff MC, Omer SB (2012). A review of fetal and infant protection associated with antenatal influenza immunization. *American Journal of Obstetrics and Gynecology*.

[62] Offit PA (2007). Thimerosal and vaccines—a cautionary tale. *The New England Journal of Medicine*.

[63] Thompson WW, Price C, Goodson B (2007). Early thimerosal exposure and neuropsychological outcomes at 7 to 10 years. *The New England Journal of Medicine*.

[64] Mrozek-Budzyn D, Majewska R, Kieltyka A, Augustyniak M (2012). Neonatal exposure to Thimerosal from vaccines and child development in the first 3years of life. *Neurotoxicology and Teratology*.

[65] (2012). Global Advisory Committee on Vaccine Safety. *The Weekly Epidemiological Record*.

[66] Ault KA, Heine RP, Riley LE (2012). Programmatic and research priorities for improving influenza immunization of pregnant women. *American Journal of Obstetrics and Gynecology*.

[67] Read JS, Riley L (2012). Progress in overcoming barriers to influenza immunization of pregnant women. *American Journal of Obstetrics and Gynecology*.

[68] Adegbola R, Nesin M, Wairagkar N (2012). Immunogenicity and efficacy of influenza immunization during pregnancy: recent and ongoing studies. *American Journal of Obstetrics and Gynecology*.

[69] Field Trial of Maternal Influenza Immunization in Asia. Identifier: NCT01034254. NCT01034254.

[70] Maternal Flu Vaccine Trial in Bamako, Mali. Identifier: NCT01430689. NCT01430689.

[71] Immunogenicity and Safety of Different Dosing Schedules of Trivalent Influenza Vaccine in HIV-infected Pregnant Women. Identifier:NCT01527825. NCT01527825.

[72] 2010–2011 Trivalent Influenza Vaccine (TIV) in Pregnant Women. Identifier: NCT01173211. NCT01173211.

[73] Influenza Vaccine in Pregnant Women. Identifier: NCT00905125. NCT00905125.

[74] Influenza Vaccine Trial in HIV Uninfected Pregnant Women. Identifier: NCT01306669. NCT01306669.

[75] Vitamin A and Maternal-Infant Flu Vaccine Response. Identifier: NCT00817661. NCT00817661.

[76] Opting In vs Opting Out. Identifier: NCT01233804. NCT01233804.

[77] Influenza Vaccination of HIV Infected Pregnant Women: Safety and Immunogenicity. Identifier: NCT01306682. NCT01306682.

